# Slightly acidic electrolyzed water as a novel thawing media combined with ultrasound for improving thawed mutton quality, nutrients and microstructure

**DOI:** 10.1016/j.fochx.2023.100630

**Published:** 2023-03-06

**Authors:** Dewei Kong, Rongwei Han, Mengdi Yuan, Qian Xi, Qijing Du, Peng Li, Yongxin Yang, S.M.E. Rahman, Jun Wang

**Affiliations:** a College of Food Science and Engineering, Qingdao Agricultural University, Qingdao 266109 China; b College of Food Science and Engineering, Tarim University, Alar 843300, China; c Department of Animal Science, Bangladesh Agricultural University, Mymensingh 2202, Bangladesh

**Keywords:** Mutton, Ultrasound-assisted thawing, Slightly acidic electrolyzed water, Quality, Nutrients, Microstructure, Thawing methods

## Abstract

The effects of ultrasound-assisted slightly acidic electrolyzed water thawing (UET), air thawing (AT), water thawing (WT) and microwave thawing (MT) on the quality, nutrients and microstructure were investigated. The UET treatment did not affect the lightness (L*) but reduced the redness (a*) and yellowness (b*) of the mutton. The UET treatment could better maintain the textural properties. The UET group had a higher immobilized water and lower free water, which was closer to the state of the control group. The UET treatment not only effectively inhibited the lipid oxidation but also reduced the loss of nutrients, especially minerals. The microstructure of the UET group was smoother and more complete, and the muscle fibers did not show significant breakage. In conclusion, UET treatment could better maintain the quality, nutrients and microstructure of thawed mutton. Therefore, UET could be regarded as a potential thawing method for application in the processing of meat products.

## Highlights

• UET treatment could improve the texture and inhibit lipid oxidation of thawed meat.
• UET treatment could reduce the loss of nutrients, especially minerals.
• The microstructure of UET group was smoother and more complete without fractures.

## 1 Introduction

Mutton is favored by consumers due to its delicious taste and nutritional value. The main nutrients in mutton include high protein, abundant minerals and vitamins, and free amino acids (Fan et al., 2021). In China, the production of mutton reached 5.14 million tons in 2021, ranking firmly among the highest in the world. The main production areas of mutton are mainly located in the northwest region of China, such as Xinjiang and Inner Mongolia (Sun et al., 2011, Wang et al., 2021). However, the consumption market of mutton is mainly distributed in the eastern and southern parts of China (Li et al., 2022). Mutton is susceptible to spoilage at room temperature because it has large water and protein contents. In addition, the presence of microorganisms and enzymes is equally not conducive to the preservation of mutton at room temperature (Wang et al., 2021). Therefore, freezing is an economical and effective method to facilitate transportation and extend the shelf life (Wang, Kong, et al., 2020b).

Thawing is a crucial step before subsequent processing or consumption (Wang, Du, et al., 2020a). The thawing process, including the thawing rate, method and temperature fluctuations, can affect the meat quality (Zhang, Liu, Xia, Sun, & Kong, 2021). A fast thawing rate had fewer adverse effects on meat product quality than a slow thawing rate (Jia, Liu, Nirasawa, & Liu, 2017). Traditional thawing methods have been applied in the home and catering industries because of their simple operation and low cost, such as air thawing, refrigerator thawing and flow water thawing (Cai et al., 2018, Lan et al., 2021). However, studies have shown that traditional thawing methods can cause irreversible damage to meat products, such as discoloration, texture softening, loss of juice, loss of nutrients and microbial growth (Guo et al., 2021), which result in further quality deterioration. In addition, Wang et al. (2020) found that temperature fluctuations can negatively affect the quality of frozen beef due to the destruction of its cells and tissues. Thus, a new and effective thawing technique was explored to overcome the damage caused by traditional thawing methods to the quality of meat products.

As an efficient and environmentally friendly technology, ultrasound has been used to thaw meat products, including chicken, yak meat, pork and various fish. Different from traditional thawing methods, ultrasound-assisted thawing can effectively enhance various mass transfer processes (Tao et al., 2022). This can be explained by the fact that ultrasound converts acoustic energy into thermal energy in food, thus increasing the thawing rate of frozen samples (Wu, Zhang, Adhikari, & Sun, 2017). The frozen tissue of the samples can absorb more energy of ultrasonic attenuation than that of the thawed tissue (Chandrapala, Oliver, Kentish, & Ashokkumar, 2013), which avoids local overheating during thawing. The cavitation effect and microjet can enhance the heat transfer between the sample and thawing medium (Sun, Kong, Liu, Zheng, & Zhang, 2021). However, with the advancement of technology, it is increasingly difficult for ultrasound treatment alone to satisfy the demand for food processing. Therefore, the combination of ultrasound with chemical factors and other emerging technologies is the development trend of the food processing industry in the future. These chemical factors mainly include slightly acidic electrolyzed water (SAEW), neutralized electrolyzed water (NEW), H_2_O_2_ and sodium hypochlorite (NaOCl), while these emerging technologies include supercritical CO_2_, high hydrostatic pressure, pulsed electric fields and ultraviolet radiation. Wang et al. (2021) found that the sample had better quality under dual-frequency sequential ultrasound thawing. Cai et al. (2019) found that ultrasound-assisted microwave thawing, and far-infrared thawing could better maintain protein secondary structure and that muscle fibers were smoother and straighter than with ultrasound thawing (UT). Ultrasound combined with plasma-activated water (PAW) thawing not only showed a good bactericidal effect but also inhibited protein oxidation (Qian et al., 2022). Ultrasound-assisted saline thawing could positively affect the quality of mirror carp (Li, Wang, Kong, Xia, & Bao, 2022). Recently, slightly acidic electrolyzed water (SAEW) has gained increasing attention from researchers because of its excellent antibacterial activity and fewer adverse effects on human health (Ding et al., 2015). Likewise, as a safe and green technology, SAEW has been used in the food industry, such as on fruits and vegetables, beef, pork and pomfret. Compared to conventional thawing methods, SAEW thawing has no adverse effects on the texture, pH and color of the meat after thawing. In addition, SAEW can better delay lipid oxidation during thawing (Liao et al., 2020). SAEW and SAEW ice could not only improve gumminess and chewiness of pomfret but reduce bacterial contamination and prolong the shelf life (Huang et al., 2021). The combined treatment with ultrasound and SAEW not only delayed color deterioration and lipid oxidation in sea bass, but also maintained better texture and sensory scores (Lan, Lang, Zhou, & Xie, 2021). In addition, ultrasound combined with slightly acidic electrolyzed water treatment can effectively inhibit protein oxidation and protect the secondary and tertiary structure of myofibrillar proteins (Kong, Han, et al., 2023a).

Based on our previous study, we found that ultrasound combined with SAEW thawing had fewer adverse effects on chicken meat quality and the myofibrillar protein structure compared to ultrasound and SAEW treatments alone (Kong et al., 2022). In addition, there are some differences in the effect of thawing methods on the meat quality due to the differences in the structure and composition of muscle fibers (O’Donnell, Tiwari, Bourke, & Cullen, 2010). Therefore, the aim was to further investigate the effect of ultrasound-assisted SAEW thawing (UET) on mutton quality, nutrients and microstructure compared to conventional thawing methods in this study. In addition, the comparison of air thawing (AT), water thawing (WT), microwave thawing (MT) and UET on the quality (pH, color, texture profile analysis (TPA), moisture migration and distribution, and lipid oxidation), nutrients (free amino acids and minerals) and microstructure of mutton was evaluated in comparison with fresh mutton (control).

## 2 Materials and methods

### 2.1 Sample preparation

The samples were all from 6-month-old Ujomuqin mutton. The samples used in the experiment were the hind legs of mutton (Inner Mongolia Zhong Ao Food Co., ltd.), whose products conformed to the Chinese national standard GB 2707–2016. The fresh mutton (4.0 kg) was obtained from a local supermarket chain (Qingdao, China). To avoid microbial contamination, samples were transported quickly to the laboratory using an insulated box with ice packs. Then, connective tissue was removed from the surface of the samples, and the samples were cut into uniform sizes and weights (3.5 × 3.5 × 3.5 cm^3^, 30 ± 5 g). The cut-mutton samples were packed using polyethylene cling film and then randomly separated into five groups. One group was not subjected to any freezing and thawing treatment and regarded as control group (fresh mutton), which was immediately tested for the indices. The other four groups were placed at −20 °C for freezing. After being frozen for 14 days, the other four groups were thawed by four different treatments (AT, WT, MT and UET).

Slightly acidic electrolyzed water (SAEW) was made through a device (Anywhere-320 W, Beijing, China) with a current of 8.0 A and an electrolysis time of 20 min. After electrolysis, the available chlorine concentration (ACC) of the SAEW was measured using a chlorine test instrument and colorimetric reagents (RC-3F, KRK Corp., Saitama, Japan). A device (PHB-1, Sanxin, China) with a pH probe and an oxidation–reduction potential (ORP) probe was used to determine the pH and ORP of SAEW. SAEW with a pH of 5.29 ± 0.01, oxidation reduction potential (ORP) of 889.67 ± 2.08 mV, and available chlorine concentration (ACC) of 42.67 ± 0.58 ppm was used as the thawing medium.

### 2.2 Thawing methods

For AT group, the samples were thawed on a plastic chopping board (35 × 25 × 0.5 cm^3^) for thawing. The samples of the AT group were placed at 10 cm intervals along a straight line on a plastic cutting board.

For WT group, the samples were separately thawed in three 500 mL glass beakers (Shuniu, Chengdu, China) containing distilled water (500 mL).

For MT group, the samples were thawed in a microwave oven (Galanz, P70D20TJ-D3, China) equipped with a turntable at the output power of 700 W and a microwave frequency of 2450 MHz. During thawing, the sample was placed in the middle of the turntable. The thawing method was intermittent thawing every 5 s interval of 10 s.

For the UET group, the samples were thawed in ultrasonic bath (KQ-500TDE, Kunshan ultrasonic instrument Co., ltd, China) containing 12.5 L SAEW. The samples were placed on the wash basket. As a power ultrasound (20–100 kHz, 10–1000 W/cm^2^) of low frequency and high intensity, 80 kHz and 300 W was used as the operating frequency and output power in the ultrasonic bath. The actual output power of 255.81 ± 8.06 W was determined using the calorimetric method.

In order to compare the effects of different thawing methods on the quality, nutrients and microstructure of meat products, the external temperature was kept within the same range during the thawing process. The sample temperature was recorded using a temperature recorder (NAPUI thermocouple, Mod. TR 230X-8, Guangdong, China) during thawing. The thawing process was completed when the sample temperature reached 4 °C.

### 2.3 pH

The pH of the samples was measured following the method of Li et al. (2022). Briefly, 10.00 g churned mutton was placed in homogenization bag containing 100 mL distilled water. The above mixture was homogenized in a bag mixer (Interscience, Bag Mixer 400, France) for 3 min. After that, the mixture was filtered using filter paper. A pH meter (Mettler-Toledo, Shanghai, China) was used to determine the obtained filtrate. Before determining the pH value of the sample, the pH meter was firstly calibrated using the two-point method. The standard buffers of pH 7.00 and pH 4.01 were used successively to calibrate the pH meter.

### 2.4 Color

A colorimeter (CR-400, Osaka, Japan), which was equipped with an 8 mm aperture, 2° standard observer and illuminant C, was used to measure the lightness (L*), redness (a*) and yellowness (b*) of fresh and thawed mutton. When the thawing process of the sample is completed, the color of six points of the sample was determined using the CIELAB system after 30 min of blooming time. The chroma and hue angle were calculated following the Eq. (1) and Eq. (2), respectively.

(1) $$\mathrm{Chroma}=\sqrt{{a*}^{2}+{b*}^{2}}$$

(2) $$\mathrm{Hue}\;\mathrm{angle}=\tan^{-1}\left(b*/a*\right)$$

### 2.5 Texture profile analysis (TPA)

The TPA of samples was determined by an analyzer (TA.XT Plus C, SMS, UK). The mutton was cut into cubes before analysis. Afterward, the sample was placed directly below the probe and compressed to 50%. The probe's moving speeds was set as 1 mm/s. The measurement interval was set to 5.0 s in two cycles of the compression test. The trigger force was set to 10 g.

### 2.6 Low-field nuclear magnetic resonance (LF-NMR)

The relaxation time (T_2_) of the samples was measured using a device (NIMI20-040V-I, Suzhou, China) to analyze the moisture mobility and content of fresh and thawed mutton. Samples (1.5 × 1.5 × 2 cm^3^) were first placed into a glass weighing bottle. Then, the glass weighing bottle was placed into a 40 mm NMR tube for measurement. The LF-NMR analysis was performed at 32 °C. Before measurement, the Q-FID sequence was used to calibrate the LF-NMR analyzer. The T_2_ of the sample was measured using a CPMG sequence. The T_2_ and corresponding area (P_2_) of each peak of the sample were obtained through MultiExp Inv Analysis software (Suzhou Niumai Analytical Instruments Co., ltd.).

### 2.7 Thiobarbituric acid-reactive substance (TBARS)

The TBARS was measured using the method of Chen et al. (2020). A minced mutton (10.00 g) was mixed into 50 mL of trichloroacetic acid (TCA) solution (7.5%, W/V). After shaking for 30 min, the mixture was filtered through a double layer of filter paper. Five milliliters of the filtrate were added to 5 mL of 0.02 M 2-thiobarbituric acid solution and then kept for 40 min at 90 °C. After that, the mixture was cooled and centrifuged (Sigma 3K15, Germany) to obtain the supernatant. The absorbance was noted at 532 nm and 600 nm. The TBARS was calculated following Eq. (3).

(3) $$\mathrm{TBARS}\left(mg/100g\right)=\frac{A_{532nm}-A_{600nm}}{155}\times 72.06\times \frac{1}{10}\times 100$$

### 2.8 Free amino acids (FAAs) content

The FAAs were determined in accordance with the method of Geng et al. (2019). Five grams (precision of 0.001 g) of mutton was mixed into 15 mL of TCA solution (5%, W/V) and then homogenized at 12000 rpm for 30 s in a homogenizer (XHF-DY, Scienzt, Ningbo, China). The mixture was centrifuged at 8000 × g to obtain the supernatant. The homogenization process and the centrifugation process of the samples were repeated two times. The supernatant was diluted to 50 mL using TCA solution in a volumetric flask. One milliliter of supernatant was placed in a vial and then dried under vacuum. The dried samples were redissolved by adding 0.02 M HCl and then filtered using a polytetrafluoroethylene (PTFE) membrane (0.22 μm). An automated amino acid analyzer (Hitachi L-8900, Tokyo, Japan) was used to determine FAAs of the samples.

### 2.9 Minerals content

The fresh and thawed samples were ground and then freeze-dried for 72 h. One gram of powdered sample (precision of 0.001 g) was placed into a digestion tube containing 10 mL nitric acid and 2 mL perchloric acid. After that, the above mixture was kept for 24 h. The samples were placed into the graphite digestion apparatus for heated digestion. The digestion process mainly included four stages: (1) The digestion temperature was gradually raised to 55 °C, 90 °C, 125 °C and 160 °C; (2) the sample digestion was completed when only approximately 1 mL of solution remained in the digestion tube; (3) the sample in the digestion tube was added to 10 mL of deionized water to remove the remaining nitric acid and perchloric acid. The sample solution in the digestion tube was diluted to 25 mL. The concentrations of K, Ca, Na, Mg, P, Cu, Fe, Mn, and Zn were determined using an Inductively Coupled Plasma Optical Emission Spectrometry (ICP-OES, Optima 8000, Perkin Elmer, USA). The concentration of Se was determined using an atomic fluorescence spectrometer (AFS-933, Beijing, China).

### 2.10 Microstructure

Samples were cut into pieces (3 × 3 × 3 mm^3^) and then fixed with 2.5% glutaraldehyde solution for 24 h. Samples were rinsed using phosphate buffer six times and dehydrated with ethanol solutions (50%, 60%, 70%, 80% and 90%). Then, the samples were dehydrated three times in 100% ethanol solution. After dehydration, the samples were replaced three times with tert-butanol. After freeze-drying, the samples were coated with gold to further observe their microstructure. The longitudinal and cross-sectional microstructures of the samples were observed at 500 × and 700 × magnifications using SEM (JSM-7500F, JEOL, Japan), respectively.

### 2.11 Statistical analysis

The data were expressed as mean ± standard deviation (SD). The three independent trials were conducted for each thawing group and control group to assess the effects of thawing methods on the quality, nutrients and microstructure of the mutton. Data from the experiments were analyzed using the compare means procedure in SPSS 19.0 software (SPSS Inc., Chicago, USA). One-way ANOVA was used to determine the significance of the main effects, and then the Duncan procedure was used to determine whether there was a significant difference between the different groups at a level of significance of *P* < 0.05. All figures were plotted using Origin Pro 2018 (Origin Lab Corporation, MA, USA).

## 3 Results and discussion

### 3.1 pH evaluation

As one of the important parameters, the pH can directly affect the color, water retention capacity and shear force (Guo et al., 2021). Changes in pH are related to the production of lactic acid and the breakdown of alkaline substances (Liu, Liang, Xia, Regenstein, & Zhou, 2013). The changes in pH of the samples are shown in Table 1. The pH values of all thawed samples were not significantly different than control group (*P* > 0.05). Therefore, the pH of mutton was not influenced by the thawing methods.

**Table 1:** The changes in pH and color of the samples under different methods.

Samples	pH	L*	a*	b*	Chroma	Hue angle
Control	5.86 ± 0.04^a^	40.93 ± 0.62^a^	17.12 ± 1.27^a^	11.79 ± 0.65^a^	20.79 ± 1.36^a^	34.60 ± 1.27^b^
AT	5.84 ± 0.09^a^	36.54 ± 1.40^b^	13.15 ± 0.91^b^	9.36 ± 0.54^b^	16.14 ± 1.00^b^	35.48 ± 1.20^b^
WT	5.93 ± 0.06^a^	41.83 ± 1.58^a^	11.41 ± 0.59^c^	9.38 ± 0.67^b^	14.78 ± 0.81^b^	39.39 ± 1.53^a^
MT	5.82 ± 0.08^a^	38.04 ± 1.32^b^	16.29 ± 1.67^a^	11.68 ± 0.81^a^	20.05 ± 1.83^a^	35.70 ± 0.89^b^
UET	5.86 ± 0.10^a^	40.41 ± 2.18^a^	12.75 ± 0.85^bc^	8.92 ± 0.62^b^	15.57 ± 0.78^b^	35.02 ± 2.60^b^

*Control, fresh mutton; AT, air thawing; WT, water thawing; MT, microwave thawing; UET, ultrasound-assisted slightly acidic electrolyzed water thawing.

*Results are expressed as mean ± standard deviation (SD). Different letters for the same index indicate significant differences.

### 3.2 Color evaluation

As an important sensory indicator, color can visually reflect the meat freshness. Generally, the color change of meat products is affected by lipid oxidation, pigment degradation, protein oxidation and water loss (Leygonie, Britz, & Hoffman, 2012). The changes in color of the samples are shown in Table 1. We found that the L* treated by AT and MT treatment would be significantly lower than control (*P* < 0.05). This can be explained by the fact that AT and MT could result in severe damage muscle structure. However, L* in the UET group did not change significantly (*P* > 0.05), indicating UET treatment could effectively protect the microstructure of mutton. The components (Cl^-^, Na^+^, Cl_2_, and HClO) of SAEW had a positive effect on structural protection during thawing. Li et al. (2022) found that the appropriate addition of salt ions could effectively protect the structural integrity of fish cells and inhibit oxidation reactions. The decrease in a* might be associated with myoglobin degeneration and pigment loss during thawing (Leygonie et al., 2012, Sun et al., 2021). The a* underwent a significant decrease in the AT, WT and UET groups (*P* < 0.05). The longer thawing time of the AT and WT treatments resulted in a higher degree of protein oxidation. In addition, the free radicals, thermal effects, and acoustic effects generated by ultrasound could damage the structure of pigments in muscle cells during thawing, resulting in a decrease of a* (Hughes, Clarke, Purslow, & Warner, 2020). The changes in b* are mainly related to lipid and protein oxidation. After thawing, the samples in the MT group had the highest b* values, which may be related to protein oxidation due to local overheating during microwave thawing. The chroma can reflect the stability of the meat. A significant decrease in chroma values occurred in the AT, WT and UET groups (*P* < 0.05), indicating that these thawing treatments were not conducive to maintaining color stability. Regarding the hue angle, the WT group had a significantly higher than control (*P* < 0.05), indicating a higher degree of protein oxidation in the samples. Protein oxidation could increase the b* value and hue angle during freezing and thawing. In conclusion, MT treatment had less adverse effects on the color of thawed meat products and its color was closest to that of fresh mutton, followed by UET treatment.

### 3.3 TPA evaluation

As an important indicator of meat quality, texture can not only reflect the freshness of meat products but also directly affect the level of consumer satisfaction. The typical indicators are used to describe the changes in texture (Table 2). The hardness of the thawed samples significantly decreased than control (*P* < 0.05). However, the hardness of the samples caused by UET treatment was closest to control, which indicated that UET treatment could delay protein oxidation. Gan, Zhang, Mujumdar, and Jiang (2022) found that the decrease in hardness was associated with cross-linking, denaturation and degradation of proteins during the thawing process. In addition, sodium chloride in SAEW had a positive effect on maintaining the meat hardness (Shimamura, Shinke, Hiraishi, Tsuchiya, & Masuda, 2016). The springiness was significantly decreased in the AT, WT and MT groups (*P* < 0.05). The decrease in springiness after thawing could be attributed to the reabsorption of water and damage to the tissue structure by ice crystals (Sun et al., 2021). For cohesiveness, adhesiveness and chewiness, the change trends of different groups tended to be consistent. The cohesiveness, adhesiveness, and chewiness of all thawing groups were significantly lower than the control (*P* < 0.05). However, the cohesiveness, adhesiveness, and chewiness of the UET group was closer to control group. The resilience of the thawed groups significantly decreased than control. The decrease in the meat texture index was associated with the destruction of muscle tissue as well as protein and lipid oxidation. Overall, the textural properties were all affected by thawing process. The WT group had the smallest texture indices among all treatment groups, which may be related to the prolonged immersion of the samples in water, indicating that the WT treatment could cause severe damage to the samples. However, the texture indices of the UET group were the closest to control, indicating UET treatment can protect tissue structure and make the qualities of thawed samples closer to those of fresh meat compared to other thawing methods. This may be related to the cavitation effect generated by ultrasound. Wang et al. (2021) found that the cavitation effect could allow more air to enter the liquid and form more cavitation nuclei. In conclusion, although the textural properties of the samples decreased to some extent after thawing, the textural properties of the UET group were the best among all thawing methods.

**Table 2:** The changes in TPA of the sample under different methods.

Samples	Hardness(N)	Springiness(mm)	Cohesiveness(–)	Gumminess(N)	Chewiness(N*mm)	Resilience(–)
Control	403.26 ± 37.39^a^	0.341 ± 0.088^a^	0.276 ± 0.029^a^	111.69 ± 18.26^a^	38.85 ± 13.83^a^	0.105 ± 0.009^a^
AT	215.46 ± 18.69^d^	0.166 ± 0.029^cd^	0.122 ± 0.020^cd^	26.49 ± 6.97^cd^	4.52 ± 2.01^c^	0.040 ± 0.008^cd^
WT	158.84 ± 11.70^e^	0.095 ± 0.004^d^	0.079 ± 0.007^d^	12.45 ± 0.31^d^	1.19 ± 0.03^c^	0.025 ± 0.002^d^
MT	264.90 ± 2.49^c^	0.198 ± 0.069^bc^	0.159 ± 0.047^bc^	42.06 ± 12.47^c^	5.54 ± 1.28^c^	0.054 ± 0.013^bc^
UET	356.27 ± 11.23^b^	0.275 ± 0.015^ab^	0.182 ± 0.017^b^	64.82 ± 6.45^b^	17.85 ± 2.23^b^	0.061 ± 0.006^b^

*Control, fresh mutton; AT, air thawing; WT, water thawing; MT, microwave thawing; UET, ultrasound-assisted slightly acidic electrolyzed water thawing.

*Results are expressed as mean ± standard deviation (SD). Different letters for the same index indicate significant differences.

### 3.4 LF-NMR analysis

**Fig. 1:**
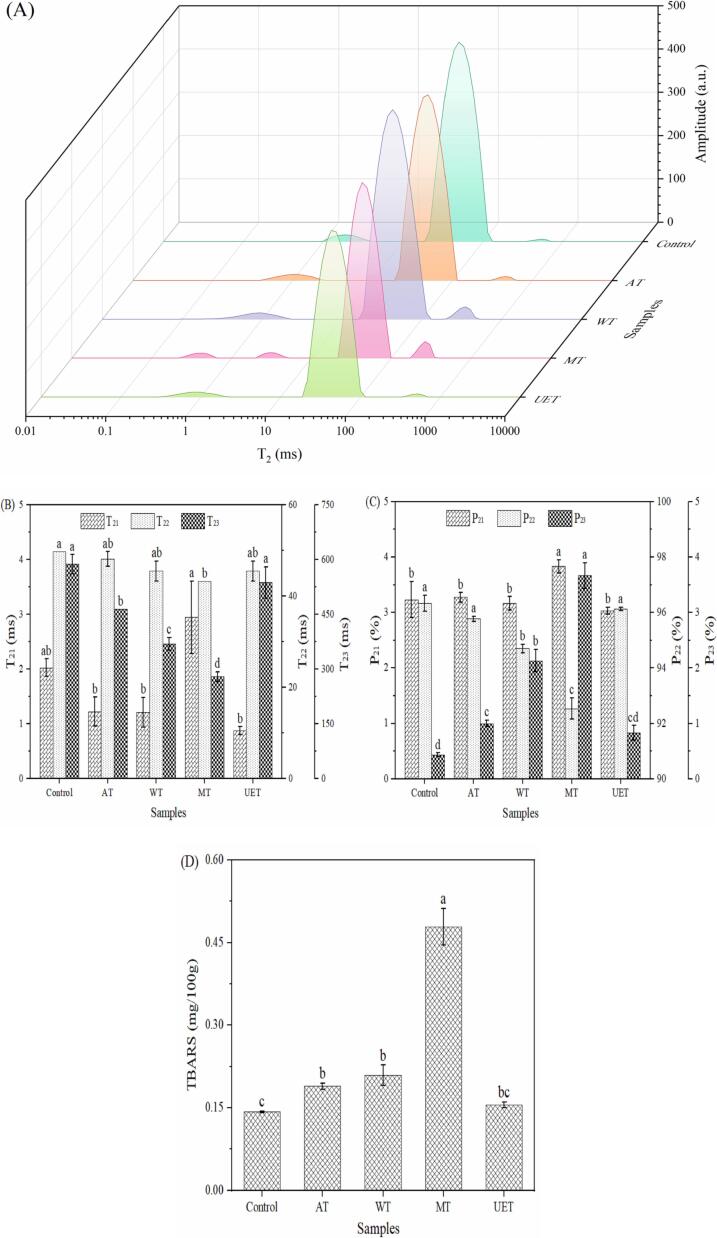
The changes in the moisture migration curve (A), T_2_ relaxation time (B) and corresponding peak area ratio (C) and TBARS (D) of the samples under different methods. Control, fresh mutton; AT, air thawing; WT, water thawing; MT, microwave thawing; UET, ultrasound-assisted SAEW thawing. Different letters for the same index indicate significant differences.

As a nondestructive spectroscopic technique, LF-NMR is currently an important tool for the analysis of moisture distribution and mobility (Gan et al., 2022). The three different peaks on the curve represent the three different states of water. The changes in the moisture migration curve, relaxation time (T_2_) and corresponding peak area ratio of the samples are shown in Fig. 1A, Fig. 1B and Fig. 1C, respectively. The longer in T_2_ relaxation time meant an increase in water mobility and redistribution (Li et al., 2022). After thawing, the T_21_ and P_21_ did not change from the control group (*P* > 0.05), except for the MT group. Bound water was not affected by thawing and freezing (Cai et al., 2019). We found that two peaks of MT group were observed between 0 and 10 ms, which indicated that MT treatment affected the state of bound water in the samples. The two peaks represent water strongly bound and weakly bound to macromolecules. A significant decrease in P_21_ values was observed in the WT and MT groups (*P* < 0.05), indicating the WT and MT treatments caused the conversion between a portion of the immobilized water and free water. However, the P_21_ values in the UET group did not change (*P* > 0.05), indicating that the UET treatment had no influence on the content of immobilized water. The T_23_ values were significantly reduced in the AT, WT and MT groups (*P* < 0.05), indicating the mobility of free water was affected by AT, WT and MT treatments. However, there was no significant difference in P_23_ values in the UET group compared to the control group (*P* > 0.05), which indicated the UET treatment had no influence on the amount of free water in the samples. This is because the ultrasound-induced cavitation effect can protect the muscle fiber structure (Kong, Quan, et al., 2023b), which reduces damage to the sample during thawing. Additionally, appropriate salt ions may help to maintain the osmotic pressure of muscle cells (Li et al., 2022), thus reducing the migration of water in the samples. The UET group had the highest amount of immobilized water and the lowest amount of free water among all thawing methods. Therefore, the UET treatment was beneficial in maintaining the water in the samples and made the state of the samples closer to that of fresh mutton.

### 3.5 TBARS evaluation

Lipid oxidation could cause undesirable quality changes, such as discoloration, flavor deterioration, and reduced nutritional value (Liao et al., 2020). The changes in the TBARS values are displayed in Fig. 1D. After thawing, a significant increase in TBARS values happened in the AT, WT and MT groups (*P* < 0.05), indicating that these treatments lead to lipid oxidation during thawing. Among them, the MT group had the highest TBARS value among all thawing methods (*P* < 0.05). Lorentzen et al. (2020) found that it was related to the excessive heat generated during microwave thawing. However, the TBARS of the UET group was not significantly different than control (*P* > 0.05), which indicated UET treatment could inhibit the lipid oxidation of meat. This may be because the thermal effect generated by ultrasound could stabilize the temperature of the samples near the freezing point during thawing (Gan et al., 2022), thus avoiding local overheating. In conclusion, UET treatment could effectively reduce lipid oxidation of the samples during thawing process.

### 3.6 FAAs content analysis

The FAAs content can affect the flavor of meat, such as the aroma, sweetness and bitterness, which are closely associated with R-group hydrophobicity of amino acids (Bian, Cheng, Yu, Mei, & Xie, 2022). Glutamic acid, alanine, glycine and aspartic acid can provide fresh sweetness, while arginine and lysine are the main sources of bitterness (Chu, Tan, Bian, & Xie, 2022). The changes and heatmap in FAAs content of samples are shown in Table 3 and Fig. S1, respectively. After thawing, the total FAAs content was significantly lower than control (*P* < 0.05), which may be associated with the disruption of protein structure during thawing (Bian et al., 2022). This could be explained by the longer time required for AT and WT treatments, which would lead to protein denaturation. Although the thawing rate of MT is higher, local overheating occurred during the thawing process, which can lead to severe protein oxidation. The cavitation effect could produce free radicals in the thawing medium, which may promote protein degradation (Kang, Gao, Ge, Zhou, & Zhang, 2017). In conclusion, the FAAs content of the samples underwent different degrees of decrease after thawing, which could be explained by the protein structure being disrupted due to the thawing process.

**Table 3:** Changes in the FAAs content of samples under different methods.

FAAs (mg/100 g)	Control	AT	WT	MT	UET
Val	0.112 ± 0.005^c^	0.108 ± 0.003^c^	0.110 ± 0.003^c^	0.132 ± 0.002^a^	0.121 ± 0.003^b^
Ile	0.049 ± 0.001^a^	0.040 ± 0.001^c^	0.033 ± 0.002^d^	0.042 ± 0.001^b^	0.034 ± 0.000^d^
Leu	0.104 ± 0.003^a^	0.074 ± 0.002^c^	0.061 ± 0.001^e^	0.081 ± 0.001^b^	0.065 ± 0.002^d^
Phe	0.096 ± 0.000^a^	0.053 ± 0.001^c^	0.031 ± 0.001^d^	0.059 ± 0.000^b^	0.050 ± 0.001^c^
Thr	0.391 ± 0.007^a^	0.090 ± 0.002^c^	0.099 ± 0.001^b^	0.091 ± 0.000^c^	0.085 ± 0.002^c^
Lys	0.093 ± 0.003^b^	0.096 ± 0.003^b^	0.085 ± 0.001^c^	0.105 ± 0.001^a^	0.088 ± 0.000^c^
Gly	0.177 ± 0.003^a^	0.161 ± 0.001^b^	0.125 ± 0.001^c^	0.158 ± 0.001^c^	0.131 ± 0.002^d^
Ala	0.669 ± 0.001^b^	0.661 ± 0.007^b^	0.556 ± 0.002^d^	0.743 ± 0.000^a^	0.644 ± 0.007^c^
Ser	0.123 ± 0.003^a^	0.081 ± 0.003^c^	0.070 ± 0.001^d^	0.087 ± 0.001^b^	0.070 ± 0.001^d^
Asp	0.021 ± 0.001^a^	0.010 ± 0.001^b^	0.010 ± 0.001^b^	0.009 ± 0.001^b^	0.008 ± 0.000^c^
Glu	0.449 ± 0.002^a^	0.390 ± 0.004^b^	0.365 ± 0.002^b^	0.379 ± 0.031^b^	0.376 ± 0.005^b^
Pro	0.054 ± 0.003^c^	0.054 ± 0.004^c^	0.043 ± 0.007^d^	0.074 ± 0.001^a^	0.064 ± 0.002^b^
Arg	0.131 ± 0.006^d^	0.193 ± 0.004^b^	0.172 ± 0.008^c^	0.222 ± 0.003^a^	0.197 ± 0.003^b^
Tyr	0.071 ± 0.007^a^	0.016 ± 0.005^d^	0.035 ± 0.001^c^	0.043 ± 0.001^b^	0.029 ± 0.001^c^
**Total**	2.541 ± 0.011^a^	2.029 ± 0.009^c^	1.795 ± 0.006^e^	2.224 ± 0.036^b^	1.962 ± 0.024^d^

*Control, fresh mutton; AT, air thawing; WT, water thawing; MT, microwave thawing; UET, ultrasound-assisted slightly acidic electrolyzed water thawing.

*Results are expressed as mean ± standard deviation (SD). Different letters for the same index indicate significant differences.

### 3.7 Minerals content analysis

Minerals are very important for human health (Guo et al., 2021). Different from proteins and amino acids, minerals cannot be synthesized in the human body. Therefore, minerals must be constantly replenished from the diet, and meat is one of the most important ways for the body to replenish minerals. Minerals can be classified as constant elements and trace elements according to their content in the body. The changes and heatmap in mineral content of the samples are shown in Table 4 and Fig. S2, respectively. Among them, K was the most abundant element in the meat, which was similar with the findings of Mohammed et al. (2021). After thawing, the K content in the AT, WT and MT groups decreased significantly (*P* < 0.05), while no change occurred between the UET and control groups (*P* > 0.05). This can be attributed to the UET group having a smaller thawing loss and faster thawing rate. In addition, we found among all thawing groups, the UET group had the highest Ca, Mg, Cu, Fe, Mn, Zn and P contents, and the total mineral content of the UET group did not change significantly in comparison with control (*P* > 0.05), indicating the UET treatment could reduce loss of mineral content of meat during thawing. Because ultrasound-induced microjets could decrease damage of ice crystals to tissue structures (Li et al., 2022). In conclusion, UET treatment could better maintain the content of minerals in the sample.

**Table 4:** The changes in minerals in the samples under different methods.

Minerals (mg/kg)	Control	AT	WT	MT	UET
K	14564.98 ± 612.64^a^	13148.65 ± 232.12^b^	10287.78 ± 842.02^c^	13261.35 ± 86.69^b^	13980.98 ± 543.77^ab^
Ca	213.43 ± 9.20^d^	219.22 ± 8.35^d^	307.14 ± 30.90^b^	264.94 ± 13.97^c^	378.96 ± 21.81^a^
Na	1891.12 ± 33.43^c^	1712.62 ± 30.98^d^	1367.27 ± 92.64^e^	2164.69 ± 41.42^a^	2079.91 ± 55.33^b^
Mg	1025.28 ± 33.24^b^	981.60 ± 19.72^b^	963.50 ± 60.21^b^	1000.84 ± 17.33^b^	1143.60 ± 46.49^a^
P	7983.66 ± 219.16^a^	7722.50 ± 171.67^a^	6113.59 ± 815.53^b^	7407.05 ± 524.44^a^	7938.37 ± 251.13^a^
Cu	5.70 ± 0.10^c^	6.23 ± 0.08^b^	5.95 ± 0.38^bc^	5.83 ± 0.25^c^	7.05 ± 0.21^a^
Fe	80.31 ± 1.49^b^	83.01 ± 5.26^b^	76.94 ± 4.71^b^	76.95 ± 2.10^b^	88.95 ± 3.89^a^
Mn	0.58 ± 0.01^b^	0.58 ± 0.03^b^	0.57 ± 0.02^b^	0.60 ± 0.03^b^	0.72 ± 0.05^a^
Zn	173.97 ± 1.65^a^	144.39 ± 2.16^c^	152.28 ± 9.26^c^	161.13 ± 5.16^b^	172.00 ± 4.82^a^
Se	0.18 ± 0.01^a^	0.12 ± 0.01^b^	0.12 ± 0.01^b^	0.12 ± 0.02^b^	0.13 ± 0.01^b^
**Total**	25939.19 ± 839.78^a^	24018.91 ± 359.56^c^	19275.14 ± 1753.25^d^	24343.51 ± 584.21^bc^	25790.66 ± 794.13^ab^

*Control, fresh mutton; AT, air thawing; WT, water thawing; MT, microwave thawing; UET, ultrasound-assisted slightly acidic electrolyzed water thawing.

*Results are expressed as mean ± standard deviation (SD). Different letters for the same index indicate significant differences.

### 3.8 Microstructure observation

**Fig. 2:**
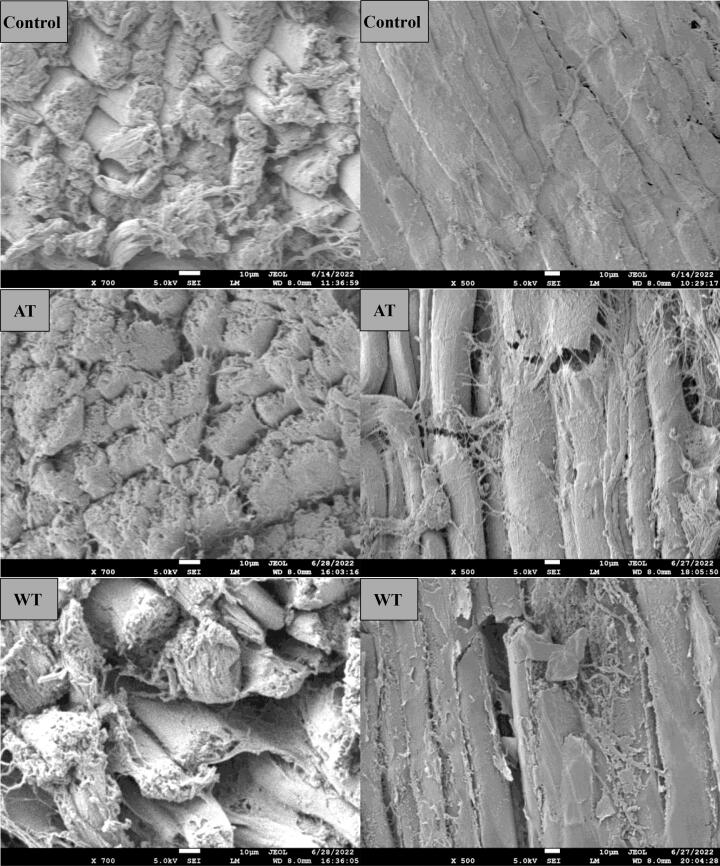

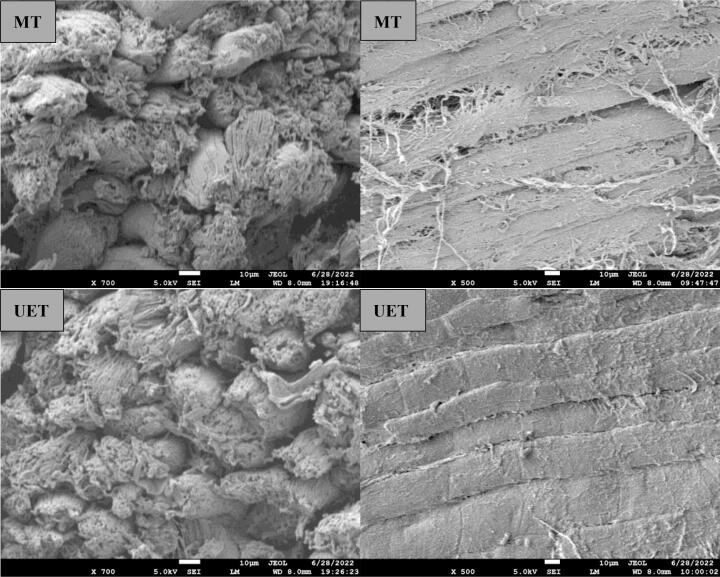
The cross-sectional and longitudinal microstructures of the samples under different methods. Control, fresh mutton; AT, air thawing; WT, water thawing; MT, microwave thawing; UET, ultrasound-assisted SAEW thawing.

The microstructure can directly reflect the effect of the thawing method on the muscle fibers. The longitudinal and cross-sectional microstructures of the samples are showed in Fig. 2. In the control group, the tissue structure was intact, with muscle fibers closely arranged with each other. The gap of the AT group was slightly larger than control between muscle fibers. In addition, the muscle fibers of the AT group showed multiple breaks in the longitudinal figure. The gap between muscle fibers of the WT group was further enlarged. Like the AT group, the muscle fibers of the WT group also showed breaks. This may be associated with the longer thawing time of AT and WT groups. For the MT group, although the muscle fibers did not show breaks, their surface was rough, and the gap was large between the muscle fibers. This may be caused by the overheating phenomenon generated by microwaves during the thawing process. Compared with other thawing groups, the microstructures of the UET group were more compact and intact. The ultrasound-induced microjets could improve mass transfer and reduce the damage of ice crystals to tissues (Li et al., 2022), thus making the thawed samples relatively structurally intact. In addition, the thawing medium SAEW could inhibit protein and lipid oxidation of the samples (Liao et al., 2020), which was similarly conducive to protecting the microstructure of the samples. In conclusion, the microstructure of the UET group was more compact and smoother, which was more similar to the control group.

## 4 Conclusions

In this study, the effects of AT, WT, MT and UET on the quality, nutrients and microstructure of frozen mutton were investigated. After thawing, the pH of the sample was not influenced by the thawing methods. UET treatment had less effect on the L* of the samples but reduced the a* and b* of the samples. According to TPA and LF-NMR, UET treatment could maintain better textural properties and reduce water migration of the samples, which made the state of thawed samples closer to that of fresh samples. In addition, UET treatment not only effectively inhibited lipid oxidation of the samples but also avoided the loss of nutrients during the thawing process. The microstructure of the UET group was relatively more intact and compact than that of the conventional thawing method. In conclusion, UET was an excellent and promising thawing method to ensure the quality, nutrients and microstructure of thawed meat products.

## CRediT authorship contribution statement

**Dewei Kong:** Investigation, Methodology, Data curation, Formal analysis, Validation, Writing – original draft. **Rongwei Han:** Methodology, Writing – review & editing. **Mengdi Yuan:** Investigation, Methodology. **Qian Xi:** Investigation, Methodology. **Qijing Du:** Methodology. **Peng Li:** Methodology. **Yongxin Yang:** Methodology, Writing – review & editing. **S.M.E. Rahman:** Methodology. **Jun Wang:** Conceptualization, Supervision, Project administration, Funding acquisition.

## Declaration of Competing Interest

The authors declare that they have no known competing financial interests or personal relationships that could have appeared to influence the work reported in this paper.

## Data Availability

Data will be made available on request.
