# Supplementary material for: Slightly acidic electrolyzed water as a novel thawing media combined with ultrasound for improving thawed mutton quality, nutrients and microstructure
Source: Food Chem X. 2023 Mar 6;18:100630. doi: 10.1016/j.fochx.2023.100630 (PMC10023902; doi:10.1016/j.fochx.2023.100630)
Supplement: Supplementary data 1 [file mmc1.docx]

**Fig. S1**. Heatmap of the FAAs content of samples under different methods. Control, fresh mutton; AT, air thawing; WT, water thawing; MT, microwave thawing; UET, ultrasound-assisted SAEW thawing.





**Fig. S2**. Heatmap of the minerals content of samples under different methods. Control, fresh mutton; AT, air thawing; WT, water thawing; MT, microwave thawing; UET, ultrasound-assisted SAEW thawing.
